# Community-based counselling programme for pregnant women with alcohol problems in Cape Town, South Africa: a qualitative study of the views of pregnant women and healthcare professionals

**DOI:** 10.3389/fpsyt.2023.1203835

**Published:** 2023-07-06

**Authors:** Petal Petersen Williams, Jodilee Erasmus, Bronwyn Myers, Abhijit Nadkarni, Daniela C. Fuhr

**Affiliations:** ^1^Mental Health, Alcohol, Substance use and Tobacco Research Unit, South African Medical Research Unit, Cape Town, South Africa; ^2^Department of Psychiatry and Mental Health, University of Cape Town, Cape Town, South Africa; ^3^Institute for Life Course Health Research, Department of Global Health, Stellenbosch University, Stellenbosch, South Africa; ^4^Curtin enAble Institute, Faculty of Health Sciences, Curtin University, Perth, WA, Australia; ^5^Centre for Global Mental Health (CGMH), Department of Population Health, London School of Hygiene & Tropical Medicine, London, United Kingdom; ^6^Addictions Research Group, Sangath, Goa, India; ^7^Leibniz Institute of Prevention Research and Epidemiology, Bremen, Germany; ^8^Health Sciences, University of Bremen, Bremen, Germany; ^9^Department of Health Services Research and Policy, London School of Hygiene and Tropical Medicine, London, United Kingdom

**Keywords:** pregnant women, alcohol, counselling, community health workers (CHWs), South Africa

## Abstract

**Introduction:**

South Africa lacks services to detect and address alcohol use during pregnancy, particularly outside of health-care facilities. This study aimed to explore pregnant women and healthcare providers’ perceptions of the acceptability, feasibility and appeal of a community-based counselling programme for pregnant women with alcohol problems.

**Methods:**

Twenty-eight in-depth interviews with pregnant women who drink, Community Health Workers (CHWs) and antenatal service providers were conducted. Transcribed interviews were analyzed thematically using a combined deductive and inductive approach.

**Results:**

Women reported feeling uncomfortable seeking help for their alcohol use at antenatal clinics, limiting usefulness of current support services. All stakeholders perceived a community-based intervention to be acceptable and feasible as it could be integrated with other CHW-delivered services. Participants thought an intervention should facilitate early linkage to antenatal services and should include partners or family members. The feasibility of an intervention may depend on the relationship between CHWs and clinic-based antenatal staff, and their relationships with pregnant women. Clinic and community challenges to implementation were raised. Clinic-level challenges included shortage of space, staff capacity, high number of pregnant women, long waiting times, financial burden of having to travel to a clinic, lack of comfort and privacy and staff attitudes. Community-level challenges included crime, lack of privacy, lack of attention given competing interests in the home, fear due to abuse, and stigma and discrimination from other community members. Suggestions for overcoming these challenges were provided.

**Conclusion:**

Findings provide essential information to facilitate the adaptation of a community-based alcohol counselling programme for greater acceptability, feasibility and cultural appropriateness for the South African context. Intensive training, supervision and support is required to ensure the programme is delivered as planned.

## 1. Background

Prenatal exposure to alcohol can cause pregnancy, neonatal and birth complications, including miscarriage, low birth weight and poor growth outcomes, stillbirth, and lifelong disorders including foetal alcohol spectrum disorders (FASD) (1–3). FASD is an umbrella term which describes several conditions associated with foetal alcohol exposure (foetal alcohol effects, partial foetal alcohol syndrome, alcohol-related birth defects and alcohol-related neurodevelopmental disorder) (4). South Africa (SA) is estimated to have the highest prevalence of FASD globally with 111 cases per 1,000 population (5); and the prevalence is particularly high (and rising) in the wine-growing region of the Western Cape of SA where estimated prevalence is 20–28% (6–9). Drinking amongst pregnant women is common, particularly in the impoverished townships of the Western Cape, where alcohol intake during pregnancy has become normalized (10). The prevalence of alcohol consumption in pregnancy, (including heavy episodic drinking) has ranged from 20–61% across various studies covering a range of measures of frequency and quantity (10–12) with approximately 20% of women who are pregnant testing positive for alcohol use by urinalysis (12).

Guidelines for the identification and management of substance use and substance use disorders in pregnancy have been developed by the WHO to provide evidence-based advice that can be applied within countries (13). Despite some efforts to address the high prevalence of FASD and the availability of guidelines for the prevention and management of FASD in SA (14), there is no national, coordinated, multisectoral effort or specific policies to address FASD (15, 16). Locally, services to detect alcohol use during pregnancy and to prevent alcohol-exposed pregnancies (AEPs) are largely absent outside of antenatal services offered through primary health care (PHC) clinics (17). Pregnant women who drink alcohol do not access these facility-based antenatal services until late in their pregnancies, with more than 75% of women accessing antenatal care for the first time during their second trimester and beyond (12, 18). Service planners in the Western Cape recognise that, to reach pregnant women at risk of having an alcohol exposed pregnancy for counselling, they need to move beyond only offering services in PHC clinics to include community-based services that reach women in their communities. This is especially important to facilitate earlier pregnancy recognition for women who have unplanned pregnancies, resulting in delayed access to antenatal services. Given shortages of health care professionals, these community-based antenatal services have been task-shared from nurses and midwives to trained community health workers (CHWs). South Africa has adopted task-sharing as a strategy for overcoming human resource shortages for health, as recommended by the WHO (19). CHWs form a bridge between communities and healthcare facilities (20). CHWs in the Western Cape are trained to provide a range of community-based health services that have historically focused on the prevention and management of infectious and non-communicable diseases (21), but have recently been extended to include early pregnancy and postnatal care (22). Whilst they undergo training in maternal and child health care, this training does not address how to recognise or address alcohol use amongst pregnant women, partly because there is a dearth of evidence-based programmes to address alcohol use during pregnancy that are acceptable to pregnant women, feasible for CHW delivery and suitable for the local context.

The current study is a first step towards developing a programme for pregnant women with alcohol problems that can be integrated into CHW-delivered maternal and child health services. This study aimed to explore pregnant women’s explanatory models of alcohol use and perceptions of the acceptability, feasibility and barriers to accessing a community-based programme for pregnant women who drink from the perspective of pregnant women who drink, CHWs and Antenatal Service Providers (ASPs).

## 2. Methods

### 2.1. Setting and recruitment

The current study is presented in line with consolidated criteria for REporting Qualitative research (COREQ) guidance (23). We conducted face-to-face and telephonic in-depth interviews with CHWs, ASPs and pregnant women who drink alcohol. Interviews explored alcohol use amongst pregnant women; barriers to service engagement; how to enhance the uptake of a proposed intervention; perceptions of acceptable and appropriate counselling delivery agents; settings in which to deliver counselling; and methods of counselling delivery (e.g., acceptability of face to face individual sessions). For the latter, we were particularly interested in the perceived feasibility and acceptability of CHW delivered screening and interventions. The topic guides are included in Annexure A-C. Pregnant women were recruited from communities in the Cape Flats region of the Western Cape province. CHWs and ASPs (i.e., midwives and nurses) who are working in the Western Cape with women who are at risk of heavy drinking were eligible for inclusion. Pregnant women were identified through outreach activities by visiting areas frequented by potential participants and which were pointed out by community leaders and CHWs who helped identify initial contacts. These women were approached by research staff who explained the study to them and if they were interested women were screened and either taken to the research site immediately to be interviewed or an appointment made for a time that was convenient for them. We then used snowball sampling from the initial contacts who identified peers who were pregnant women who drink alcohol. Through this peer-driven social network sampling all women identified by peers and who were approached accepted to take part. The pregnant women were required to be 18 years or older, report heavy drinking (defined as at least 1 day of drinking more than 4 drinks in the last month) measured using the Alcohol Timeline Follow Back, and screen positive for hazardous alcohol use (Alcohol Use Disorders Identification Test (AUDIT) score ≥ 8) (24).

### 2.2. Procedures

Interviews were conducted by the second author and project-coordinator (JE) who is female and has a post-graduate degree and more than 5 years qualitative research experience. JE had no prior relationship with study participants. Interviews were audio-recorded before being transcribed verbatim and lasted up to 60 min. Interviews took place between December 2020 and March 2021. Participants were reimbursed ZAR100 [~US $5.42 (ZAR18.45/$1)] for their time.

### 2.3. Analysis

NVivo 12 was used to store data and facilitate analysis. Results were analysed thematically using the Braun and Clarke approach (25). Data were analysed using a deductive approach to coding based on the research questions, which was combined with an inductive approach to allow other emergent themes to be identified. The second author (JE) conducted the initial process of familiarisation through a review of transcripts and coding. The first (PPW) and second author discussed the initial framework. After coding of initial transcripts, they discussed refining of codes and initial themes. Coding then continued of all interviews. Any coding disagreements were resolved through discussion and consultation with the last author (DF). Following this, themes were reviewed, defined and named as per Braun and Clarke guidelines.

### 2.4. Ethical approval

The study was approved by the South African Medical Research Council’s (SAMRC) ethics committee (EC006-4/2020) and further approval from the Western Cape Health Department (WCDoH) (WC_202006_046) to conduct research with healthcare professionals was granted. All participants provided consent to participate in the study. Women were provided with referrals for available services. Face-to-face interviews were conducted at CHWs and ASPs place of work in a secure and private room. Face-to-face interviews with pregnant women were conducted in a secure and private room. For telephonic interviews with pregnant participants (*n* = 2), both were asked to provide a convenient time for the interview to be conducted and these interviews occurred when the two participants were alone. Confidentiality and anonymity were assured in that all data would be aggregated before presentation and no names mentioned.

## 3. Results

The results are classified into three major themes: alcohol use and pregnant women; barriers to accessing care; and perceptions of a counselling programme for problem alcohol use in pregnancy.

### 3.1. Sample characteristics

In total we conducted 28 interviews (*n* = 5 CHWs; *n* = 8 ASPs; and *n* = 15 pregnant women) before data saturation was reached, in line with recommendations for thematic analysis (26–28). The five CHWs were employed by an NGO that delivers comprehensive wellness services in response to communicable diseases. The ASPs consisted of eight midwives working in four PHC facilities, with the majority of women accessing these services being socio-economically disadvantaged. All CHWs and ASPs were female. The mean age of the pregnant women was 24.9 years (range 19–41 years), one was Black African and 14 were coloured (of mixed-race ancestry). AUDIT scores ranged from 9 to 30 with a mean score of 18.1 (SD = 5.9) and 10 (67%) scoring in the likely dependence range (AUDIT ≥15).

### 3.2. Alcohol use and pregnant women

#### 3.2.1. Subtheme 1: extent and perceived causes of alcohol use amongst pregnant women

Participants discussed the prevalence of alcohol use amongst pregnant women and factors influencing drinking behaviour. The CHWs confirmed that during their work they often encounter pregnant women who drink alcohol. Reasons for drinking ranged from stressors related to not being able to financially provide for their families, troublesome romantic relationships, to ignorance about the effects of alcohol during pregnancy.

*Especially for the adults also that’s drinking, maybe they have a problem in the house, you know like money, food and stuff like that. Now you rather go drink, to forget that the children need this (CHW003).*

*You know how it goes, sometimes your boyfriend or husband makes you angry or there are problems like the fact that he does not work or there is no income, then you might feel like taking your last money and going drinking. (P011).*

Participants described similar reasons for why pregnant women drink, with relationship difficulties, intimate partner violence, and ambivalence about the pregnancy being the most salient amongst these.

*If they want to spite the boyfriend and say, I want to hurt your child or so, now I’m going to show you that I’m going to drink. I do not want the child, I want it to come down [miscarry]. People talk like that, just because they see that the boyfriend has another girlfriend (CHW001).*

#### 3.2.2. Subtheme 2: acceptability of alcohol use in pregnancy

CHWs reported that at a community-level alcohol is not recognized as a harmful substance or viewed as negatively as other substances therefore normalizing its use.

*Yeah, it’s like in the community everybody is labelled because you are doing drugs but nobody is labelled because they drink… like I said people now do not really see it as a problem (CHW005).*

Pregnant participants reported a range of drinking patterns, with only a few drinking less since pregnancy recognition indicating its acceptability. However, although drinking was common, all the pregnant participants viewed their alcohol use as unacceptable and thought they should be drinking substantially less.

*During the week I do not even think about that, but when Friday comes by, Saturday morning I’m stressed out, tired and I’m kinda like a glass of wine and then it will be a second one, third one and then it ends up being 5 litres. It’s just that I cannot control it, if I could I would stop (P006).*

#### 3.2.3. Subtheme 3: knowledge of impact of alcohol use in pregnancy

Many pregnant women shared the correct information and expressed concern about how alcohol use could affect their unborn infant. However, some pregnant women lacked this understanding or were indifferent to the effects of alcohol.

*Yes, because I was drinking when I had my first and second babies and they came out normal, so why must I quit if my children came out normal? (P007).*

*No, I’m not that concerned. I feel life inside of me every day, it’s not that it’s quiet, so I feel happy (P009).*

### 3.3. Barriers to accessing care

#### 3.3.1. Subtheme 1: alcohol use as a barrier to service engagement

Participants described alcohol use as a barrier to antenatal service engagement. ASPs shared their experiences of late initiation of antenatal care and/or irregular attendance of scheduled appointments by pregnant women who drink alcohol. They reported how some women who drink arrive at the clinic when they are in labour without any prior antenatal care. They also described instances where women arrived at the labour ward drunk claiming to be in labour, but once checked there were no signs of labour which they believed due to their intoxication. They reported that these incidences place additional burden on the clinic staff, especially where they are understaffed, and have low resources. According to ASPs, these limited resources affect their ability to provide pregnant women who drink with alcohol-related health education and to refer women for alcohol-related services.

#### 3.3.2. Subtheme 2: relationships with service providers

ASPs felt that pregnant women who drink were fearful of attending their clinic sessions, especially when they knew any of the nurses in a personal capacity. Staff working in PHC facilities often reside in the same area where they work.

*Stigma in that they do not want to come because this person knows them here, that person knows them (ASP005).*

CHWs shared reports from pregnant women who experienced negative reactions from nurses about their alcohol use during their antenatal clinic visits. They thought that these stigmatising interactions hindered some pregnant women from accessing services.

*Yes. How do I say it – it is not good to judge and shame people in public; “Look how you look.” “You’re smelling.” That will not work. Or if the child is underweight; “You did not breastfeed them properly” (CHW001).*

Pregnant women admitted to not disclosing their alcohol use during their health screening as they anticipated and feared negative reactions from nurses. Nurses acknowledged that their style of communicating with pregnant women who drink was not ideal.

ASPs described trying to instil fear and scare patients in the hope that they will either disclose their alcohol use or change their drinking. As one pointed out:

*So you have to interrogate the hell out of them. Because normally what they tell you is not what it seems. They will not tell you that they drank last night but you can smell it so I’m like no but you did drink… I do not let up. I’m relentless. Many other people will just like leave it. I’m relentless. I will ask you. If you go into labour now and that water broke and it smells like alcohol do you know your baby can die (ASP004).*

#### 3.3.3. Subtheme 3: partner relationships

All three groups of participants described relationships with romantic partners as a barrier to care. According to ASPs, these relationships often influence pregnant women’s use of antenatal services, especially where these relationships were violent.

*And also you talk about the gender-based violence to them because others they are abused at home that is why maybe they are not going to the clinic, or else they are depending to the alcohol. Things like that (ASP001).*

#### 3.3.4. Subtheme 4: alcohol screening and referral for treatment

It was apparent that screening and referral for alcohol use was not a rigorous process. ASPs described alcohol screening as being part of the mental and health screening process at the first appointment and consists of only one question: “Do you drink alcohol?” If a patient responds “No,” then the nurse is not able to move forward with a referral for alcohol-related services. Even if the patient discloses alcohol use, ASPs are only able to offer a referral if a process is in place. All ASPs reported providing a 5-min information session on healthy pregnancy that addresses FAS, if a pregnant woman discloses alcohol use. However, they did not clarify whether this alcohol education was provided to women who did not disclose alcohol use.

ASPs felt that pregnant women were not always forthcoming about their alcohol consumption, either denying any alcohol use or underreporting their consumption. Given the reliance on self-disclosed alcohol use for referral, this was viewed as a barrier to offering appropriate and effective assistance to pregnant women who drink.

*The patients lie. The patients do not say the truth. They will say they do not drink, but then they do. But we do not have any proof. So, we have to take the word as they say it, and most of them do not admit to alcohol (ASP005).*

Similarly, CHWs shared that pregnant women’s nondisclosure of alcohol use affected their ability to refer women for additional care. Whilst CHW service delivery did not include routine screening for alcohol use, they did describe offering education and information on the dangers of drinking whilst pregnant if they suspected alcohol use.

CHWs also described some difficulty in getting pregnant women to accept help for their alcohol use. They thought this was due to a lack of trust in available services and support.

*“I do not need health education. I can cope and raise my child on my own. And my child will be fine.” They do not understand that we are just here to support and be on standby, not to judge them (CHW001).*

Pregnant women considered the screening process at antenatal services as problematic. They reported receiving very little to no education on guidelines for alcohol use during pregnancy or counselling for alcohol-related difficulties.

*They said they cannot tell me not to drink, but I should know my limits. I told them that it’s only 2 to 3 beers a day (P010).*

*There was no counselling, nothing!… Yet on my maternity book it states that I needed counselling yet I did not get it. (P003).*

### 3.4. Perceptions of a counselling programme for alcohol use in pregnancy

All three groups of participants were asked their opinion on a counselling programme for alcohol use for pregnant women and their perceptions of the feasibility and acceptability of CHW-delivered screening and interventions. As an example, we summarized counselling for alcohol problems (CAP), a lay-delivered community-based intervention to reduce alcohol use (29). CAP is a manualized brief psychological therapy based on motivational interviewing and behavioural techniques such as problem-based management and cognitive behaviour therapy. It is currently the only available evidence-based alcohol-focused brief therapy delivered in community settings by lay counsellors in a low– and middle-income country (LMIC) (29, 30).

#### 3.4.1. Subtheme 1: feasibility and barriers to accessing a community based programme for pregnant women

Despite raising some anticipated delivery challenges, all three groups responded positively and confirmed the need for such a programme. CHWs discussed the opportunities they have to include counselling for pregnant women who use alcohol and the feasibility of being delivery agents. CHWs shared the opportunities they have to impact early pregnancy recognition and to refer pregnant women to antenatal services and other support services where these are available.

Pregnant women reflected on how a counselling programme would benefit them, especially as they had no support for alcohol-related difficulties.

*Because why they going to be in a programme, so this programme it will be almost like a rehab, now these things that you going to give us to do in the house and so (P001).*

#### 3.4.2. Subtheme 2: acceptability of and recommendations for a community based programme for pregnant women

All participants reacted positively to the content of a community-based alcohol programme, and pregnant women particularly liked the idea of having CHW support for healthy choices during pregnancy.

*That will be great idea to have someone whom you can come to, help you with solution in life, at the end of the day that person is needed for better decisions, coming with better ideas and solutions… Of course other women will love this, I know most of them they are bored at home and they would love conversations that are helping and sometimes you need to take up a different challenge (P003).*

Whilst a few participants thought an intervention could be clinic-based, the majority thought that the community would be the ideal implementation setting for such an intervention. Participants did however note that CHWs would need to consider whether women’s homes are safe and empowering settings for interventions, highlighting the prevalence of gender-based violence. These factors would need to be taken into consideration when deciding the location or venue for services.

*I think it’s a good thing but then again, it depends on the community you work in, like a lot of the time the abuser is in the home. And that woman is not going to open up… but then this person might discourage this woman also to not come to this place (CHW002).*

Participants all thought there should be multiple intervention sessions ranging from twice a week to twice a month, up to four to six times, over several months of the pregnancy. Suggestions for the duration of the sessions ranged from as short as 5 min, up to 2 h. Whilst ASPs thought midwives could deliver the intervention, CHWs and pregnant women felt CHWs were best placed as the delivery agent. There were mixed views about whether the intervention should target all pregnant women (so as not to miss those who deny drinking) or be restricted to those women who report alcohol use.

Several clinic and community challenges to implementation were raised. Firstly, clinic-level challenges included shortage of space, staff capacity, high number of pregnant women, long waiting times, financial burden of having to travel to a clinic, lack of comfort and privacy and staff attitudes. Community-level challenges included crime, lack of privacy, lack of attention given competing interests in the home, fear due to abuse, and stigma and discrimination from other community members. Suggestions for overcoming these challenges included providing individual sessions rather than group sessions, using whatsapp groups for support and communication, providing vouchers and also something to eat and drink at the sessions and the addition of peer support groups. Finally, participations recommended the inclusion of additional intervention content related to breastfeeding, family planning, termination of pregnancy, recreational activities, exercise, nutrition and healthy eating and life skills training. All participants thought intervention content should be reinforced through the provision of information pamphlets or an intervention booklet.

Table 1 summarises the proposed components of a community-based alcohol programme based on what all three groups of stakeholders consider relevant, appropriate, and feasible to deliver (see Supplement 1 for *more detail on this stakeholder feedback*).

**Table 1:** Proposed elements of a community-based intervention for pregnant women who are drinking.

Intervention component	Proposed for intervention
Venue	5 individual sessions in the home, monitored by progress, and extended if necessary
	Pregnant women move on to Support group for the rest of their pregnancy
How often?	Once a week for 5 weeks (max over 8-week period), with homework between sessions.
For what period?	Individual sessions 5 weeks – to be completed over an 8-week period if needed (exploration of final session as group)
Time taken for each session?	Individual sessions: 30–60 min; support group sessions 2–3 h
Level of drinking	Individual sessions for all pregnant women who report drinking above recommended guidelines in pregnancy
Challenges to the clinic	Challenges mentioned: space, staff capacity, high number of pregnant women, long waiting times, financial burden of having to travel to clinic
Facilitation of implementation	Making use of the following: Whatsapp groups A certificate of completion after each session; to monitor their own progress Pregnant women to sign a document as a commitment to the programme for themselves and baby. Engaging activities and games at support group Involving external service providers such as Dietician, dentistry etc. to provide service at support group Provision of food and refreshments Include a small gift once entire programme is complete (Month before due date)
Additional components	Mental health Use of other substances, and over the counter/prescription medications Family planning Breastfeeding Wellbeing of baby Safe exercises routines Nutrition – how to eat well on a low budget. Arts and crafts Development of a skill (eg: making something for your baby) Significant other to attend the first session – providing them with a brochure and guiding them on how to be supportive and as someone for the pregnant women to be accountable to
Perceived acceptability of programme	Pregnant women, CHWS and ASP, were generally positive and supported the overall programme. Pregnant women were accepting of the involvement of a ‘significant other’, on the condition that it is not mandatory, as often, they do not have a sober-living partner, or family member who they think could support them.
Resources	Making use of pamphlets for the significant other, and a manual that they can take home, and go through themselves, a drinking diary to monitor their progress

## 4. Discussion

Alcohol use amongst pregnant women is perceived by healthcare providers to be widespread and pregnant women themselves report high levels of drinking despite having some insight into the negative impact of their drinking on their unborn infants. Several barriers to accessing care were identified in the current study including the influence of alcohol consumption on antenatal service attendance and a shortage of resources preventing adequate screening, education and referral for alcohol use. Findings suggest that alcohol use during pregnancy is under-detected due to inadequate screening processes. There is a need to reevaluate screening processes and procedures so that more time is spent on building rapport and an enabling environment in which women feel comfortable disclosing alcohol use. In keeping with other studies (31–33), emphasis was placed on non-disclosure by pregnant women being the reason for the lack of care pregnant women with alcohol problems receive. It has also been suggested that the absence of a formal alcohol screening and intervention protocol in antenatal services impacts on the care that pregnant women who drink receive (31). Similarly, referral systems and support structures were described as lacking in healthcare facilities and in the community. This is not surprising as other studies have highlighted the lack of referral pathways for patients attending primary care clinics who disclose problems related to alcohol use (34, 35). Unclear or non-existent guidelines for referring women for non-medical reasons related to alcohol use limits the extent to which women are able to access the services they need. There is thus a need to formalise a referral pathway and to develop and formalise processes for how to refer patients to these services as part of a programme of care for women who drink in the community.

It was noted that staff attitudes and approach may be preventing effective care and service provision. Changing the messaging from one that is confrontational and condescending, to one that is motivational, and non-judgmental is what pregnant women would deem a safer environment for them to comfortably disclose their alcohol use. The role of stigma on alcohol disclosure and on access to health services and how stigmatising attitudes from health providers towards patients who drink and the impact this has on health service use and alcohol outcomes has been highlighted in earlier studies (36, 37). Amongst pregnant women, studies have shown that fear and stigma may hinder women from accessing antenatal and other health services or disclosing substance use (38). Whilst medical assessments and procedures were reported as adequate, caring inter-personal relationships and interactions were reported to be lacking. Providing SPs with training on relationship and trust building and how to create safe spaces is crucial. CHWs noted that current activities and model of service provision enables the inclusion of counselling for alcohol-related problems. Being able to integrate the proposed activities into an already existing structure in the community would save costs and would also advance the skills development of the staff. CHWs can additionally be upskilled to provide some of the additional intervention components recommended in this paper.

Importantly, this counselling programme would need to be offered as part of a whole systems approach to supporting women with alcohol problems. As a brief therapy comprising at least five sessions and offering ongoing monitoring and support for alcohol change, it is best suited for women with AUDIT scores <20 (in keeping with WHO guidelines) (24). We acknowledge that additional services will be needed to support women with higher AUDIT scores indicative of alcohol dependence. With limited access to and lengthy wait times for specialist alcohol services in South Africa and no services tailored to the needs of pregnant women (39, 40), we propose delivering this counselling programme as part of a stepped care approach to treatment. In this model, women with AUDIT scores indicative of dependence are referred for specialist services but have an opportunity to access and benefit from this community programme whilst they wait to access specialist care.

Given the workforce constraints in low-resource settings, task-sharing approaches where non-specialist workers such as CHWs deliver evidence-based psychosocial interventions for alcohol use are being promoted as feasible, acceptable and cost effective (41, 42) and supported by South Africa’s health policies (43). Case studies from other parts of Africa and Asia similarly demonstrate that CHWs are well positioned geographically and socially to deliver some aspects of maternal and newborn health (MNH) (44) and alcohol interventions [37]. The proposed programme may therefore be relevant in settings similar to SA where lay counsellors are positioned to deliver psychosocial interventions. CHWs shared many challenges they currently face in their day to day activities, which mirrored the challenges they preempted from the proposed programme. Challenges such as lack of privacy, safety in the home of the pregnant women, unsafe areas, and engaging with women who are not forthcoming in advancing their relationship with the CHWs. These challenges need to be addressed in developing a counselling programme for pregnant women with alcohol problems, and CHWs themselves made recommendations for how to overcome some of these barriers. They recommended that a programme of care for pregnant women with alcohol problems is delivered in women’s homes and that change in alcohol use is monitored. The opportunity for early referrals to antenatal clinics and early initiation of antenatal care exists and will increase overall wellbeing of mom and baby.

Contrary to other studies conducted in the same region where pregnant women were largely unfamiliar with FASD or had limited knowledge of the impacts of drinking during pregnancy (45), many pregnant women in the current study were informed about the effects of alcohol use in pregnancy. Therefore, interventions need to address psycho-social struggles, particularly related to experiences of stress, interpersonal relationships and experiences of violence and move beyond information sharing and education to support women to reduce their drinking. As reflected in the suggested additional components, addressing mental health and providing a holistic integrated programme of support to pregnant women with alcohol problems, such as the desired sessions on family planning, breastfeeding and nutrition, exercise, and skills development, may lead to improved retention in antenatal care, and improved maternal and child outcomes.

This is the first study that sought to assess the potential feasibility and acceptability of a counselling programme for pregnant women with alcohol problems in the Western Cape. We could capture the experiences of women with regards to their drinking and we were able to assess current barriers to accessing services from a range of stakeholders. However, there are some limitations to consider. First, there may have been social desirability bias particularly from ASPs and CHWs who may be invested in seeing a programme developed to prevent drinking in pregnancy. Second, it is possible that some recall bias related to the extent of alcohol consumption may have been present. Third, given the inclusion criteria, all pregnant women were drinking at hazardous or harmful patterns. Since no drinking in pregnancy is recommended, it may have been useful to include participants reporting any use of alcohol rather than using general population cut-off points on the AUDIT to guide recruitment. This inclusion of heavy drinkers only may bias acceptability, which may not be generalizable to all pregnant women who drink. Finally, given the peer-driven recruitment, findings are limited to a particular social network of pregnant women with problem alcohol use.

## 5. Conclusion

Findings from the formative research are being used to design a counselling programme for pregnant women with alcohol problems that may be acceptable, feasible and culturally appropriate for the local context. These findings will inform the development of an intervention protocol and field manual to be tested in a feasibility study of the relevance, appropriateness and acceptability of the programme.

## Data availability statement

The raw data supporting the conclusions of this article will be made available by the authors, without undue reservation.

## Ethics statement

The studies involving human participants were reviewed and approved by South African Medical Research Council’s (SAMRC) ethics committee. The patients/participants provided their written informed consent to participate in this study.

## Author contributions

PP and DF conceptualized the study. JE conducted the qualitative interviews. PP and JE conducted the analysis and PP prepared the manuscript first draft. BM and AN helped develop and refine the study and all authors revised the draft versions of the manuscript critically. All authors contributed to the article and approved the submitted version.

## Funding

This study was funded by the National Research Foundation of South Africa (SRUG190404427201). The research was also supported by the South African Medical Research Council.

## Conflict of interest

The authors declare that the research was conducted in the absence of any commercial or financial relationships that could be construed as a potential conflict of interest.

## Publisher’s note

All claims expressed in this article are solely those of the authors and do not necessarily represent those of their affiliated organizations, or those of the publisher, the editors and the reviewers. Any product that may be evaluated in this article, or claim that may be made by its manufacturer, is not guaranteed or endorsed by the publisher.
