# Supplementary material for: Community-based counselling programme for pregnant women with alcohol problems in Cape Town, South Africa: a qualitative study of the views of pregnant women and healthcare professionals
Source: Front Psychiatry. 2023 Jul 6;14:1203835. doi: 10.3389/fpsyt.2023.1203835 (PMC10357510; doi:10.3389/fpsyt.2023.1203835)
Supplement: Supplementary file 1 [file Table_1.DOCX]

**Supp 1**

***Inputs related to components of CAP for pregnant women who are drinking alcohol***

| **Suggested Components of the CAP-Pre programme** | **Antenatal service providers** | **Community Healthcare Workers** | **Pregnant Women who drink alcohol** |
| --- | --- | --- | --- |
| **Venue** | *Clinic: I would say antenatal would be the best because they start with your booking. (ASP001)*  *Clinic: What I'm saying is we can start with a group session. And then we can call the individuals (ASP003)*  *Community: But most of them, they won't tell you if I'm drinking. They will say no a long time ago…. Maybe if someone can interview about that question or maybe at home, then she can get the answers. Then you group those patients.*  *(ASP006)*  *Community: You see if you can get them in the community already. Like I said if you can educate them in the community, then they can embark on their health before they get to us. (ASP005)*  *Community: It will work in the community because remember the Patients don’t at all book early. So if they can be caught before they book, it will be good. Because now we're actually waiting for them to one day come to the clinic while damage is being done.*  *(ASP005)* | *Clinic: We can work hand-in-hand like that. For example if you come to book for pregnancy, it’s just not bookings, there’s a counselling session also for substance abuse whether you drink or not. But everyone is – not to say forced – make it compulsory for you to go for that counselling (CHW001)*  *Community: We begin at their homes and then later we can move to start a support group (CHW001)*  *Community: Then if you go to them, they feel like you respect them or they have that thing then they want to hear what you will say about this (CHW004)* | *Community: Because there will be a lot of people that I can communicate with whereas at home I’m alone (P010)*  *Community: Maybe in the community hall…In a group (P005)*  *Community: think it should be in the community hall... Then we can talk about why we drink a lot. Like for instance I've been drinking since I was 15 years old (P006)*  *Community: I think at my house, because I don’t know if I’d be able to walk far. My feet are swollen due to the pregnancy. (P009)*  *Community: It won’t be a problem if they come to my house… rather do it alone and to discuss this with my family. (P011)*  *Clinic: Because it’s safer and you can talk if you want to talk. (P011)* |
| **How often?** | *And if they are working you make a time maybe one Saturday once a month. (ASP002)*  *Maybe two per week (ASP001)*  *Maybe once a week, and then we will see the outcome. Then you can change the time to fewer then, fortnightly or monthly. (AS003)*  *If it's a normal low risk pregnancy, they come four to six times. (ASP008)* | *About once a week. (CHW001)*  *Maybe like once week. (CHW002)*  *Maybe once or twice a month (CHW005)* | *However, they do it, I will do it. (P001)*  *An hour and a half that’s a good time (P003)*  *Twice a month. (P005)*  *Like Saturdays in the afternoon, when everybody is at home(P006).*  *We can do it twice a month (P009)* |
| **For what period?** | *And that you have to walk with them all that 40 weeks, you walk with that patient. For 40 weeks (ASP001)*  *I think we should start this maybe 5 months onwards. (ASP003)*  *Will depend when you started how many you take because those health community workers are going to see that we need the extra time for this. (ASP001)*  *I think four is enough.*  *(ASP005)* | *Might say after the four sessions that you feel like this person might reach her goal that she set for her. But we’re not going to know maybe a month or two maybe down the line, is it still there. Isn’t there something that to always keep – I’m not saying keep an eye on this person but maybe keep her for that time or period (CHW002)*  *And maybe for an hour or two hours to see how it works for the first session. If you see oh the four sessions really works, then you can maybe extend it or what. (CHW003)* | *Maybe 4 to 5 months. (P010)*  *I think it must go on forever because even when the baby is there you'll still want to drink.*  *(P006)*  *Four months (P005)*  *Maybe two months or when people can decide that they have learned a lot and can go home (P007)*  *Maybe 3 or 6 months (P011)* |
| **Time taken for each session?** | *I think about 1 hour (ASP001)*  *20 minutes to half an hour (ASP005)*  *About a 5-minute session (ASP008)* | *Say like a half an hour but something like that. But if you say now like the additional sessions, it should depend on the person and the one that you are dealing with. Somebody might need maybe one extra session or two but it depends on the situation (CHW002)*  *30 minutes because some people have got problems more than the others (CHW005)* | *Two hours (P008)*  *Maybe from like in the morning till 3pm (P010)*  *30 minutes (P005)*  *50 minutes to an hour (P006)*  *I’d probably prefer a half hour at the most (P009)*  *Maybe one or two hours (P011)* |
| **Delivery Agent** | *I would say the midwife. Most Midwives won't agree with me because they feel as they've got such a lot going on already, there's so much that we must do. We can't take on another added responsibility as well.. our time is so limited we can't spend so much time one-on-one with the patient. They normally skel [shout] me and say I talk too much (ASP004)* | *Yes, it will really help in my work, improve the standard of my work and I will be able to do more for the community (CHW001)* | *Because most of them (CHWs) talk to you, as if they know you a long time…They know how to work with you…it’s best because they understand the people better than what the nurses (P015)*  *I choose the community healthcare worker because I want to talk to someone… I must trust them and they must trust me (P014)* |
| **Level of drinking** | *…if you say only for those who are drinking you won't find them because they won't tell you if they are drinking (ASP006)*  *No, if you are focusing just on them, then anyone any age any amount of alcohol (ASP005)* | *For every pregnant woman (CHW002)*  *I would say we can start with those who are just starting drinking (CHW003)* | *Heavy drinkers (P008)*  *I think it must be for all women who are drinking (P010)*  *It should be for all. Pregnant women who drink as well as those who abuse medicine (P009)* |
| **Challenges to the clinic** | *The problem will start there. Who is going to do this? You can talk to the staff about this. You can raise whatever you want to. Secondly and thinking of the space because we have a shortage of space. That’s the main thing*  *(ASP003)*  *There is no space, so that's also a barrier because the counsellors don't have a room where they can work in. They must share rooms.*  *(ASP005)*  *I think it's transport if they are coming here.*  *(ASP006)*  *Like I said even we have a lot of talks, but like I said when they sit and wait, they're not really interested. They just want to be in and out, especially people abusing alcohol and drugs…they don't want to be there for the whole day at the clinic. (ASP008)* | *If that were something, it should be by a place where it’s more relaxing, it’s not like “I need to go now.” It’s like the clinic, nobody likes to go anyway to a hospital. It’s like so formal set-up, man. (CHW002)*  *They don’t even want to go in the clinic, so at all it wouldn’t work (CHW004)*  *People will tell you here; “I don’t even want to go to the clinic because I don’t even have money to travel there.” And it’s too far to walk (CHW002)* | *It’s too much, there are a lot of people coming in and out, so I think we won’t have our privacy (P010)*  *If we do it by the clinic the nurses are they don’t know what to do or what they doing they just there to monitor their days, they just there for schedules so that they can get paid, they don’t care about us (P003)*  *Because pregnant women go a lot in clinics for checkups and they stand in long queues. So they will be like, not again (P007)* |
| **Challenges to the community** | *And also you talk about the gender-based violence to them because others they are abused at home that is why maybe they are not going to the clinic, or else they are depending to the alcohol. Things like that… but in these days you are working under a lot of crime for door-to-door (ASP001)*  *You must make it an event if you want to have it in the community. And if you want to have follow-up sessions you must hope and Pray they come. Because I know we tried once. We ended up with 4. It's not about alcohol. But we ended up with 4 and we started with about 20 pregnant women (ASP008)* | *Then we stand at the gate and say, if you feel uncomfortable or feel you can’t speak, can I make an appointment with you to speak at your front gate? You ask and you don’t have to do it in front of the family (CHW001)*  *Their cooperation. If I get there and we made an appointment to do the session and they don’t open the door or they’re not interested or they do the session and they’re distracted and don’t take note of what I’m saying and I’m trying to help. Or a family member will say get done, you must still clean the house, you understand (CHW001)*  *Men are so … especially when they are abusing and this person Is drinking now … Because here there also goes a lot of abuse but then this person might discourage this woman also to not come to this place (CHW002)*  *Community hall: will be like stigma and discrimination. That is one thing that will be there. Because the group is telling her, I don’t like you because of certain things. Now I won’t come. So maybe two or three persons who maybe come from different areas but now from one area. Because rather put it from different areas, they don’t know each other. (CHW003)*  *People want my help, they do but they are not going to talk or disclose in a group especially that I know are living in the same area, I’m going to close up, I am not going to talk. Because I can see it’s not going to be confidential, somehow (CHW005)* | *Like some of people that I know they’re more of privacy than others and more for themselves (P010)*  *Some people they don’t have houses to live in, maybe they live in poverty, most of them maybe they won’t be comfortable with how their houses look or having nothing to offer when you coming in, like coffee or something to drink (P003)* |
| **Facilitation of implementation** | *I think a group session is nicer because it's more interactive. But on a personal level, maybe an individual session will be because it's more private. There's more privacy involved in an individual session. And people will think it's more serious if it's done in an individual session because this lady is speaking directly at me. But in a group session she's speaking in general to all of us.* *It doesn't feel it's directed at me specifically (ASP005)*  *I think if you give them something like vouchers, definitely they will come (ASP006)*  *And maybe if you can use WhatsApp, if they want to ask something, then you have a group*  *Of WhatsApp...(ASP006)*  *We have a dietitian also here. She usually comes and she gives them in-service training. And then we have on the dental side also, inviting Landi to give them also health Education regarding teeth, and oral health which is very important for pregnant mums (ASP007)* | *I think if you want to start I think the best would be to start individually, see how they feel individual with that, before you start the groups (CHW003)*  *Make them sign something? (CHW001)*  *Maybe it can take her mind off that alcohol. She is physically busy with her hands. Show her things that maybe you don’t need the alcohol. Show her a different side. Yes, that alcohol might have thought it was a comfort for you (CHW002)*  *Like these gardens that they started … I mean it was at least it was vegetables. I’m not saying a garden, I’m just saying what I can as a mummy do to maybe not depend on this person every time to eat for a meal and I must also eat healthy (CHW002)*  *They will come, if you have something that they can eat, providing that (CHW003)*  *Food parcel because they are unemployed*  *(CHW005)* | *Check-ups, scans so we don’t have to go to the clinic in those life long lines, maybe games. That’s if it will be in the club, or we can play games with our counsellor when they visit (P003)*  *With a cup of tea and biscuits (P006)*  *For my situation I wouldn’t mind like a pregnancy club, ladies speaking to one another, telling each other’s personal stories and experience, share all the challenges we facing individually, encourage one another. Like if things are not going well at home that could be a place you get to be happy, at the end of the day you don’t want to be stress as it’s going to affect the baby (P003)* |
| **Additional components** | *Obviously this is based on mental health as well because there's a component of that. I think that would be 80% of this program is the mental health aspect of it. Because most of the ladies drink because they have social issues at home, and mental problems… So I think empowering women to be the best kind of person that they can be (ASP004)*  *Breastfeeding... Family planning as well. If you are interested, termination of pregnancies early pregnancies so that we can also include that*  *(ASP003)*  *And arts and craft thing, something else for them to do besides drinking (ASP004)*  *It's not just the mother. So they go to the family on a whole (ASP007)* | *Some sort of exercises especially for them and obviously the way of eating healthy (CHW004)*  *And prevention also, with contraceptives and everything out there… I would like to add like a life skill or something in there…(and) I would love the drugs. ..and nutrition (CHW005)* | *Those who use drugs as well (P008)*  *Use of medicines and maybe drugs (P009)*  *Exercise is the kind of thing that keeps your mind at ease (P006)*  *What happens when you use alcohol, smoke or do drugs and how it will effect the baby (P011)* |
| **Perceived acceptability of programme** | *It would be acceptable. It will be acceptable if we talk in a nice manner, and tell her why (ASP002)*  *I think they will work. As I said some are stubborn. Do you pay for my alcohol? (ASP002)*  *So yes it would work. It would work. It sounds like a beautiful program, and I think it will work with the right angle and the right approach leading up to the introduction of this program (ASP004)* | *Some will really take the advice or help from us. But then you get people who will say; why must I do that. The negativity that they will have about it (CHW003)*  *They have great respect for the uniform. From the gangster to the elderly, and the elderly (CHW005)* | *I don’t think so…they love the wine too much (P008)*  *I would like to learn that and have someone to help me through these things (P010)*  *So, the positives is that it is in the community, women will stop drinking and they will know that there is support for them (P003)*  *Of course other women will love this, I know most of them they are bored at home and they would love conversations that are helping and sometimes you need to take up a different challenge (P003)*  *It's definitely something I want to be part of (P007)*  *Yes, but I want to drink my 8 beers, yes (P008)* |
| **Resources** | *I think pamphlets that they can read (ASP006)* | *Most of the things are also important, like a pamphlet. Like they can see. That we can explain to them but they can read it and they can see what is alcohol doing to their children (CHW003)* | *I think it will be good to give the mother a book to take home. If she takes a drink she can tick off the day she was drinking and the day she stopped (P007)* |
